# Editorial: Skull and craniofacial development and regeneration

**DOI:** 10.3389/fphys.2024.1398107

**Published:** 2024-04-08

**Authors:** Jeremie Oliver Piña, Johannes W. Von den Hoff

**Affiliations:** ^1^ Section on Craniofacial Genetic Disorders, Eunice Kennedy Shriver National Institute of Child Health and Human Development, National Institutes of Health, Bethesda, MD, United States; ^2^ Department of Biomedical Engineering, University of Utah, Salt Lake City, UT, United States; ^3^ School of Dentistry, University of Maryland, Baltimore, MD, United States; ^4^ Section Orthodontics and Craniofacial Biology, Department of Dentistry, Radboud University Medical Centre, Nijmegen, Netherlands

**Keywords:** craniofacial, regeneration, development, morphogenesis, skull

Craniofacial morphogenesis involves the complex interplay of cell adhesion molecules, epigenetic regulators, transcription factors, overlapping signaling pathways, and mechanotransducive forces to coordinate the most intricate anatomy of the human body, the head (Pina et al., 2023). Disruption of these morphogenetic cues during development by genetic and environmental factors can lead to congenital craniofacial structural anomalies, such as orofacial clefts, tooth agenesis, and craniosynostosis (Leslie et al., 2017; Oliver et al., 2020b). In this Research Topic, we are pleased to highlight an original article from Cabrera Pereira et al. unveiling the region-specific role of a gene encoding a LIM-domain homeodomain transcription factor (Lmx1b), which plays a key role in patterning the cranial mesenchyme into bone and sutures. Intriguingly, this study also found that two key sutures in the skull—the coronal and the sagittal suture—are derived from unique embryological stem cell niches. Such findings may lead to more targeted preclinical study models and therapeutic solutions to appropriately harness stem cell type-specific function and localization *in vivo*.

Mechanotransduction is a process by which mechanical stimuli are converted into biochemical signals through specific mechanisms, and this results in the activation of downstream signaling pathways with specific effects on cell behavior. Increasing interest has evolved in this area concerning development and regeneration. We highlight in this Research Topic a mini-review article from Lin et al. on the critical role of mechanical stimulation to guide cells and tissues in the craniofacial skeleton. Specifically, the roles of mechanosensitive Piezo1 and Piezo2 ion channels in craniofacial bone, tooth, and periodontal tissue are succinctly discussed, presenting the latest relevant evidence with implications for potential treatments and managements of dental and orofacial diseases and deformities.

Another original article in this Research Topic presents novel insight into the transcriptional localization of key signaling effectors and modulators in the Wnt pathway —*Wnt10a*, *Sost*, and *Dkk1*—to better understand the molecular interaction to bring about tooth organ morphogenesis. High-resolution spatial expression patterns, corroborated by single-cell RNA-sequencing of whole tooth organs, shed novel light on the regulatory mechanisms involving Wnt signaling during development in this context. Taken together, these findings shed novel light on critical craniofacial developmental processes, which may lead to the development of preclinical therapeutic models for further discovery and future clinical innovation.

After prenatal development, cells and tissues in the craniofacial complex can regenerate after traumatic injury or surgical resection of pathology (Oliver et al., 2021). Such a process requires an inflammatory cascade followed by angiogenesis, leading to mesenchymal stem cell (MSC) differentiation and functional maturation (Oliver et al., 2020a). In line with such orchestrated regenerative processes in the body, a review article from Behara and Goudy discuss an FDA-approved immunomodulatory molecule, FTY720, which has been found to locally increase pro-regenerative immune cell phenotypes (neutrophils, macrophages, monocytes), vascularization, cell proliferation and collagen deposition in preclinical injury models in diverse tissue sites. They further present evidence that the application of FTY720 using a biomaterial has demonstrated that local delivery of FTY720 promotes local wound healing leveraging an immunomodulatory mechanism. This work represents an in-depth analysis on the potential applications of FTY720 in regenerative wound healing, including its likely suitability for use for craniofacial specific soft tissue wounds.

While soft tissues are found throughout the craniofacial complex in the form of muscle, skin, and mucosa, the underlying skeletal architecture provides the bedrock of support for the aesthetic and functional characteristics intrinsic to this region of the body. In this Research Topic, a review article from Soares et al. uncovers some of the unique characteristics of the craniofacial skeleton—in particular, the mandible—and how these characteristics may hold the key to unlocking novel clinical therapies for mandibular pathology, such as osteonecrosis of the jaw (ONJ). Given the prevalence and burden of such pathologies, this work is of crucial importance as the field of craniofacial biology continues to unveil unique therapeutic solutions to alleviate the associated morbidity experienced by affected patients.

We are thrilled to showcase the highlighted works in this Research Topic of *Frontiers in Physiology* in the section on Craniofacial Biology and Dental Research. This diverse, robust work further establishes the need for more preclinical and clinical studies to push the boundaries of clinical care for patients affected by craniofacial anomalies.
